# The role of men in abandonment of female genital mutilation: a systematic review

**DOI:** 10.1186/s12889-015-2373-2

**Published:** 2015-10-08

**Authors:** Nesrin Varol, Sabera Turkmani, Kirsten Black, John Hall, Angela Dawson

**Affiliations:** Sydney Medical School, Discipline of Obstetrics and Gynaecology, University of Sydney, Sydney, NSW 2006 Australia; Centre for Midwifery, Child and Family Health, Faculty of Health, University of Technology Sydney, Sydney, NSW Australia; Centre for Clinical Epidemiology & Biostatistics, School of Medicine and Public Health, Faculty of Health and Medicine, University of Newcastle, Newcastle, NSW Australia

**Keywords:** Female genital mutilation, Men, Beliefs, Attitudes, Behaviour, Intervention programs, Systematic review

## Abstract

**Background:**

Men in their roles as fathers, husbands, community and religious leaders may play a pivotal part in the continuation of female genital mutilation (FGM). However, the research on their views of FGM and their potential role in its abandonment are not well described.

**Methods:**

We undertook a systematic review of all publications between 2004 and 2014 that explored men’s attitudes, beliefs, and behaviours in regards to FGM, as well as their ideas about FGM prevention and abandonment.

**Results:**

We included twenty peer-reviewed articles from 15 countries in the analysis. Analysis revealed ambiguity of men’s wishes in regards to the continuation of FGM. Many men wished to abandon this practice because of the physical and psychosexual complications to both women and men. Social obligation and the silent culture between the sexes were posited as major obstacles for change. Support for abandonment was influenced by notions of social obligation, religion, education, ethnicity, urban living, migration, and understanding of the negative sequelae of FGM. The strongest influence was education.

**Conclusion:**

The level of education of men was one of the most important indicators for men’s support for abandonment of FGM. Social obligation and the lack of dialogue between men and women were two key issues that men acknowledged as barriers to abandonment. Advocacy by men and collaboration between men and women’s health and community programs may be important steps forward in the abandonment process.

## Background

FGM is a transnational public health, human rights, and gender injustice issue, which more than 125 million girls and women in 29 countries of Africa and the Middle East have been subjected to [1]. It is also prevalent in some countries of Asia and migrant communities in Europe, the USA, Australia and New Zealand [1]. Even if the worldwide decline in FGM is maintained at current rates, population growth means that about 196 million girls would be cut by 2050 [2]. We therefore need a change in our approach to the prevention of this practice that can have a devastating impact not only on girls and women, but can adversely affect men [3] and communities as well.

FGM refers to all procedures involving partial or total removal of the external female genitalia or other injury to the female genital organs for non-medical reasons [4]. It is usually performed on girls from birth to age 15. Girls may die at the time of cutting from haemorrhage or infection, or experience significant physical, psychological and sexual complications [5–8]. There is a discrepancy between the wishes of many men and women to stop FGM and the reality of it continuing due to the deeply entrenched sense of social obligation to cut one’s daughter [1]. Moreover, this practice persists due to the lack of open dialogue between men and women, and reluctance to debate it in the public sphere. This precludes opportunities for culturally sensitive and critical introspection by communities [1].

Although women appear to be at the forefront of the perpetuation of FGM, there is some evidence that men may play a significant role in its continuation as fathers, husbands, and community and religious leaders [9, 10]. Existing FGM research involving men in regards to their influence on the decision-making process is very limited. There is no data on the success of involving men in the abandonment process. Moreover, there is little knowledge regarding the implication and effect of FGM practice on men. Footbinding of girls in China, a practice with similar sociocultural underpinnings, was abandoned and advocacy by men had played a crucial role [11].

Our systemic review examines perceptions and attitudes of men towards FGM, and their perceived and actual role in the abandonment process. The results have implications for research and intervention programs to empower men, women and their communities to be able to make the decision to abandon FGM.

## Methods

A textual narrative synthesis was undertaken involving the analysis of study characteristics, context, and findings [12]. A PICOS question was developed to guide this review [13]. We therefore sought to answer the question “For men who were born in countries, or claim ancestry from ethnic groups where FGM is practised, what are their attitudes, beliefs, and behaviours in regards to FGM, its prevention and abandonment?” Observational studies, quasi-experimental and non-experimental descriptive and qualitative studies were considered appropriate for inclusion in the review. If intervention studies were available, we sought to examine strategies that had led to change in knowledge, attitudes and behaviours. However, we also sought to identify the current views of men across different settings and contexts to gain insights that may provide opportunities to garner men’s support for the prevention and abandonment of FGM.

**Fig. 1:**
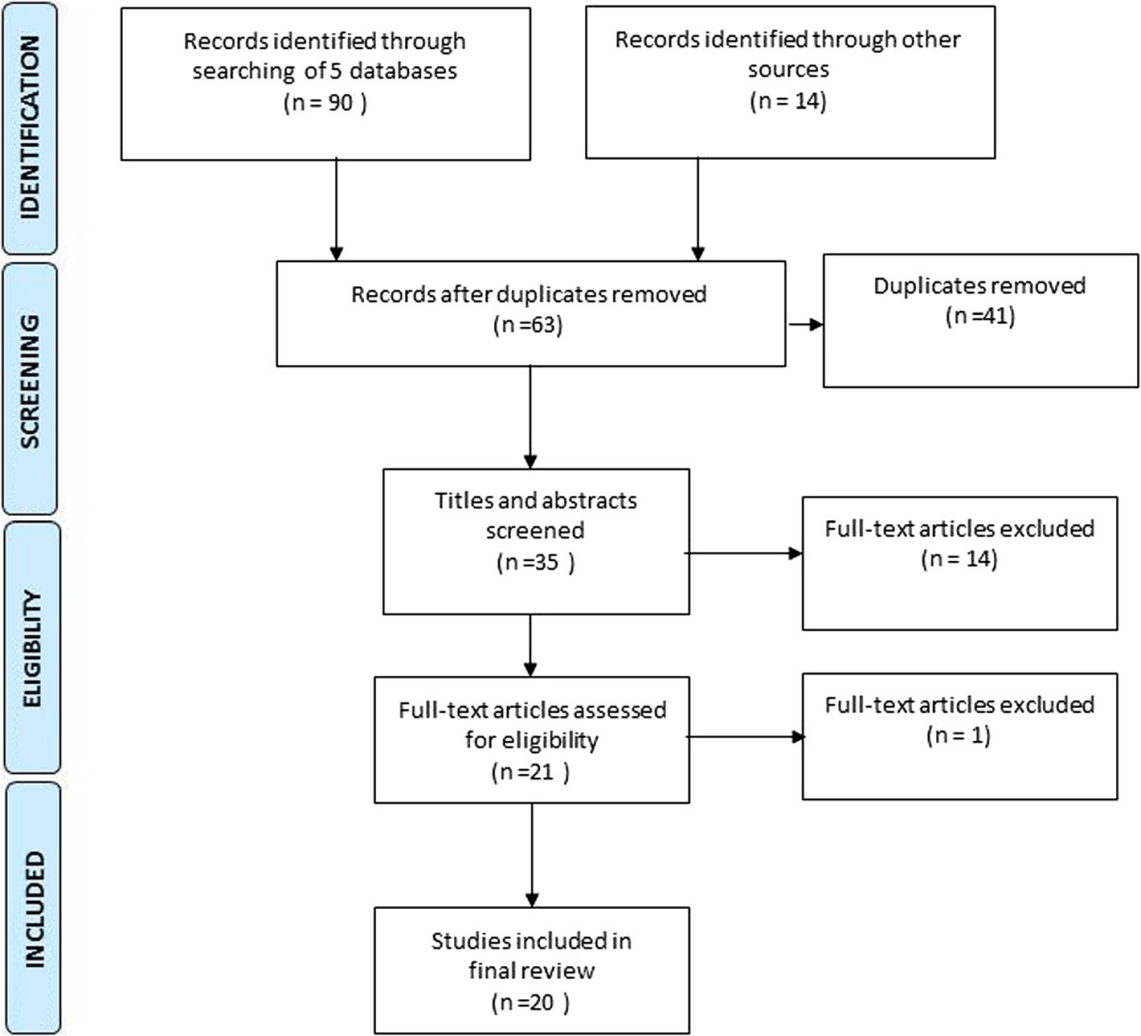
Preferred reporting diagram for systematic reviews and meta-analyses (PRISMA) showing selection of publications for review

A systematic search of the peer reviewed research literature published in English from 2005 to 2015 was undertaken by AD. The PRISMA guidelines were applied to the review process (Fig. 1) [14].

We searched Academic Search Complete (EBSCO) that included the pertinent databases Medline and CINAHL. We also searched ProQuest Health & Medical Complete, SCOPUS, Web of Science and Science Direct. The following key words were used in the search: “female genital mutilation” OR “female circumcision” OR “female genital cutting”, AND “men” AND “attitudes” OR “beliefs”, OR “behaviour”. In addition, we hand searched the reference lists of relevant papers to gain additional documents. Duplicate records were removed as well as papers that were not within the scope of the review, or older than 2005. AD and NV then screened 35 papers and removed those that did not disaggregate data by sex or gender or where male views did not provide a substantial contribution to the findings. For example two papers included only one reference to men’s understandings of FGM [15, 16]. In the paper by Shell-Duncan et al [15], it was difficult to extrapolate men’s knowledge and views from women’s. In another paper that was removed, women spoke about FGM and men did not contribute data on FGM in the study [17].

Twenty-one papers deemed eligible for inclusion were then appraised for quality using checklists to assess both qualitative and quantitative papers [18, 19]. One paper was discarded, as it was not a research study [20]. The characteristics of all 20 papers were summarised (Table 1) to examine context, sample, study aims and findings. All findings were then analysed and the data pertaining to men only were extracted. These findings were then synthesised to answer the review question as described by Harden et al [21] and key categories developed concerning men’s perceptions, issues and support. These findings were discussed by NV and AD and agreement was reached.

**Table 1:** Summary of literature in the review

Reference	Context	Method	Sample	Aim	Findings
Abdelshahid and Campbell (2015) [31]	Egypt, rural communities in the Al Qalyoubeya governorate in the Nile Delta region and Benisweif governorate	Individual semi-structured in-depth interviews	Five fathers who had at least one daughter, all Muslim, age 22-60 years	To identify psychosocial factors that shape parents’ decisions to circumcise or not circumcise their daughters	Fathers acknowledge negative impact of FGM on marital sexual relationships. Female circumcision reflects the pride of parents. The practice is linked to a daughter’s maturity and acquisition of her feminine identity. Non-circumcision is considered an obstacle to daughters’ marriage opportunities. Men believe girls who are not circumcised are promiscuous. FGM safeguards their daughter from engaging in adulterous relationships after marriage. Although lack of sexual response is inconvenient and disturbing to the husband, the practice is still deemed important to ensure marriage fidelity and retain the husband’s feeling of security regarding his wife’s fidelity. Parents find it difficult to deviate from the tradition in fear of rejection from the community. They continue to do it even though they may believe it is against their religion. They long for change but are held back by social pressure.
Al-Khulaidi et al. (2013) [22]	Yemen	Demographic Health Surveys, 1997 and 2003	Husbands of women aged 15–49, *n* = 4897 and *n* = 5908 in 1997 and 2003, respectively	To highlight attitudes of women of reproductive age (15-49) and their husbands towards the practice of FGM, and their association with the performance of FGM upon their daughters	More husbands thought FGM should be stopped in 2003 than in 1997 (*p* < 0.001). The percentage of couples that both agreed that FGM should be continued was reduced from 27.5 % in 1997 to 19.6 % in 2003 (*p* < 0.001). Husbands of women who had undergone FGM were supportive of FGM continuation, i.e. 60.1 and 49.5 % in 1997 and 2003, respectively. Both women and their husbands were more supportive regarding performance of FGM when the women had not undergone FGM (57.5 % and 47.4/%). When husbands of women with and without FGM did not agree with FGM, daughters were less likely to receive FGM in 1997 and in 2003. Women and husbands’ attitudes to not support continuation of FGM were significantly associated with not having performed FGM on their daughters, regardless of women’s age and education. A daughter was more likely to receive FGM when the attitude towards FGM of her father was positive.
Asekun-Olarinmoye and Amusan (2008) [41]	Nigeria, Shao Community	Intervention study using a multistage sampling technique – pre-tested, structured questionnaire. Intervention stage consisted of health education sessions on FGM and its complications. Survey was supplemented by in-depth qualitative interview of traditional excisors.	159 and 181 men pre- and post-intervention, respectively	To determine level of practice of FGM and impact of a health education intervention	A greater proportion of men than women did not want FGM to be stopped in the pre-intervention stage of the study, but this proportion decreased significantly (from 53.5 to 25.4 %) in the post-intervention stage. Educational status, age and gender were statistically significantly associated with whether respondents had their daughters excised or not, while religion was not.
Berggren et al. (2006) [32]	Khartoum State, Northern Sudan	Descriptive and explorative study based on qualitative interviews with men and women between September 2002 and June 2003	Ten in-depth interviews with men of age between 28 and 47, all Muslim, of varying economic status and ethnic group	To explore men’s and women’s perceptions and experiences of FGC* with emphasis on reinfibulation (RI) after delivery	Both men and women felt they were victims of consequences of FGC. Men described their own complications, male sexual dissatisfaction, compassion for female suffering and perceived challenges to their masculinity. The psychological problems created by FGC increase women’s reluctance to discuss the issue. Men claimed that it was not until they were newly married that men experienced the irrevocable consequences of their wives’ FGC. Almost all men had had sexual experiences with uncircumcised women. Men explained they tried to compensate for the effects of FGC with other means of sexual stimulation. Almost all men stated they did not want their daughters to undergo FGC and no man wanted his daughters to undergo infibulation. None of the men considered RI to be his decision, but rather one of the mother, aunt or midwife. Men stated they had only limited influence. They believed primary FGC would decrease in the future, because men were starting to look for brides who had not undergone FGC. Men mentioned the silent culture between the sexes as one of the major obstacles for change.
Diop and Askew (2009) [23]	Rural areas of southern Senegal	Quasi-experimental, pre- and post-intervention longitudinal design with a comparison group; Village Empowerment Program included classes on human rights, problem-solving process, basic hygiene, women’s health	373 men pre-intervention in 2000; 85 men participants in intervention, 198 men nonparticipants in 2002; 82 men participants, 195 nonparticipants in endline in 2003; 184 men in baseline and 198 men in endline comparison group	To evaluate effect of community education program on community members’ willingness to abandon FGM/C**	Among all groups of men, fewer men intended to cut their daughter at endline. The change was greatest among the program’s participants (from 66 to 13 %) and least among men living in the comparison villages (from 78 to 56 %). At endline, men who had participated in the program were the least likely (20 %), and men in the comparison group the most likely (63 %) to express a preference for a woman who had been cut. Three-fourth of male participants indicated they would be willing to ask people in their community to end the practice and would support women calling for the abandonment of FGM/C. In the comparison group, less than 30 % of men indicated the same.
Fahmy et al. (2010) [33]	Egypt, two rural communities	Focus group discussions and qualitative interviews	99 men, including community and religious leaders, circumcisers, and health providers; over and under 35 years of age, Muslims and Christians, educated and non-educated	To examine the role of female sexuality in women’s and men’s continued support of FGM/C, and their perceptions of its sexual consequences	Men were concerned that women’s sexual pleasure was reduced, yet were worried that uncut women would be too sexually demanding, endangering their control over the sexual relationship. Almost all men and religious leaders stated that women had as much right to enjoy sex as men. Many religious leaders understood the complexity between sexual desire and circumcision. Many men complained about problems with their sexual lives in marriage.
Gage & Van Rossem (2006) [24]	Guinea	Descriptive quantitative: secondary analysis of cross sectional survey (Demographic Health Surveys)	1851 men aged 15-59, 41 % of men were never married; 84 % Muslim; men completed 3.7 years of schooling, two-thirds of participants lived in rural areas	To examine gender differences in attitudes toward the discontinuation of FGC and gain insights into factors supporting its elimination	53 % of men reported social approval as an advantage of FGC, 42 % believed FGC reduced likelihood of premarital sex, 61 % believed it was accepted by their religion, 38 % of men opposed the continuation of FGC, 51 % of men said FGC should continue. With each additional year of schooling, the odds of favouring the discontinuation of FGC increased substantially. Urban residence increased men’s odds of supporting discontinuation of FGC. As the number of perceived disadvantages increased, respondents became more likely to support the discontinuation of FGC. Islam was not a significant variable in any of the models.
Gele, Bente, & Sundby (2013) [34]	Somalia, Hargeisa and Galkayo districts	Qualitative descriptive study using unstructured interviews	11 Somali men aged ≥18	To explore the attitudes of Somalis to the practice of FC***	Almost all men supported continuation of FGM, mainly Sunna form, while rejecting Pharaonic type. Some men were aware of health implications of the latter and perceived there to be no complications with the Sunna type. It was believed to be a religious requirement. Abandonment process was preferred to come from communities rather than government and NGOs.
Gele, Bo, & Sundby (2013) [25]	Somalia, Hargeisa district	Descriptive, quantitative, cross-sectional study; structured questionnaires	108 men	To examine attitudes of Somali men towards FC	96 % of men preferred to marry circumcised women. However, 85 % preferred the Sunna form, 11 % preferred the Pharaonic form and only 2.8 % would choose uncircumcised women to be their wives. Only two men supported the discontinuation of all forms of FC. 96 % of men perceived FC to be a religious requirement. About 90 % of men knew about complications of FC. Men were divided on whether it prevented premarital sex, led to trustable marriage and preserved the dignity of girls.
Gele, Kumar, Hjelde, & Sundby (2012) [35]	Oslo, Norway	Qualitative descriptive study using focus groups and interviews	17 men under and over 25 years of age, majority secondary school-level education, few had college or university education	To explore the attitudes of Somalis living in Norway towards FC	Almost all men (*n* = 16) expressed their rejection of all types of FC. They had high knowledge of adverse health outcomes for women and men. Majority agreed that the practice was a traditional culture as opposed to religious practice, and that it reduced female sexual pleasure. There was no pressure to do it in Norway and it was prestigious not to be circumcised there. Men explained that many people in Somalia disliked FG but continued it due to social pressure. One man had believed FC prevented sexual violence towards girls.
Jaffer et al. (2006) [26]	Oman, secondary schools	Descriptive quantitative cross sectional survey; self-administered questionnaires	Nationally representative secondary school-based sample of 1670 boys, mean age 17.3	To examine the knowledge, attitudes and practices of Omani adolescents towards reproductive issues	Nearly 80 % of adolescent boys considered FGC to be necessary and important. This attitude was significantly higher in interior regions than the capital or coastal regions and was inversely associated with higher level of parent education, especially mothers’ education. Fifty-three percent and 28 % of boys were aware of the physiological and emotional puberty changes in boys and girls, respectively.
Johnsdotter et al. (2009) [36]	Sweden	33 semi-structured qualitative interviews, snowballing sampling and contacts with immigrant organisations	33 Ethiopian and Eritrean men and women, aged 28 to 69, almost as many men as women were interviewed; majority of men were Muslim	To explore attitudes toward FGC from a migration perspective	With the exception of two men, all men strongly rejected FGC. Many men emphasised a loss of ability by women to experience sexual pleasure. Men perceived FGC as devoid of meaning.
Johnson-Agbakwu et al. (2014) [37]	Somali refugee community in Maricopa Country, Arizona, United States	Community-based participatory research, involving qualitative interviews and focus group discussions	Somali-born male refugees over age 18	To examine perspectives of Somali men toward FGC and women’s childbirth experiences in a refugee community in the United States	Men expressed concern about lack of knowledge on FC by doctors. They felt responsible to advocate on her behalf of their wives and be cultural educators to healthcare providers. Men acknowledged strong matriarchal support of FGC. All but one man disagreed with the practice. They were aware of FGC-related morbidity. Men maintained they just agreed to the practice because they did not want to upset the mothers.
Kaplan, Cham et al. (2013) [38]	Gambia	Transversal descriptive study using face-to-face questionnaire	993 men, mean age 36.5, 96 % Muslim	To explore knowledge and attitudes of Gambian men towards FGM/C, as well as practices in their family and household; to promote knowledge on FGM/C and empower communities	72 % of men did not know FGM/C had negative impact on health. Awareness of health problems was higher among younger men who were less supportive of practice. These men also had lower intention of cutting their daughters and highest willingness of seeing men intervening in prevention. Minority of men partook in decision-making, especially if they were not married. There were ethnic differences as to whether FGM/C was considered requirement by Islam.
Kaplan, Hechavarria et al. (2013) [27]	rural areas of The Gambia	A cross-sectional descriptive study with quantitative methodology	40 medical students from the Community-based Medical Programme	To examine knowledge, attitudes, and practices regarding FGM/C among health care professionals (HCP) working in rural settings in The Gambia	A significant proportion of Gambian HCP working in rural areas embraced the continuation of FGM/C (42.5 %), intended to subject their own daughters to it (47.2 %), and reported having already performed it during their medical practice (7.6 %). Their knowledge, attitudes, and practices were shaped by sex and ethnic identity. HCP belonging to traditionally practicing groups were more favourable to the perpetuation and medicalisation of FGM/C, suggesting that ethnicity prevailed over professional identity.
Mitike and Deressa (2009) [28]	Somalia Regional State, Eastern Ethiopia	Community-based cross-sectional study	246 men, Somali refugees, in three refugee camps, all Muslim	To determine the prevalence and associated factors of FGM	More men (89 %) than women (55 %) positively viewed the usefulness of anti-FGM interventions. Fewer men (75 %) than women (91 %) had the intention to cut their daughters. Participation of the parents in anti-FGM interventions was statistically associated with lower practice and intention to cut their daughters.
Ouldzeidoune et al. (2013) [29]	Mauritania	DHS of Mauritania, 2000-2001	2,191 men aged 15 and 59	To investigate factors related to FGM and gavage practices and attitudes in Mauritania; to explore implications related to the protection of children’s rights and welfare	The overall prevalence of FGM was 77 % but varied depending on ethnicity. The majority of female and male respondents favoured continuation of the practice (64 and 70 %, respectively). Men and women in rural areas were more likely to approve of continued FGM. There was discordance between male and female beliefs that the opposite sex desired the continuation of the practice, with 37 % of women reporting that they though that males wanted to continue and 55 % of men reporting that they believed women wanted it to continue.
Ruiz et al. (2014) [39]	Spain	Descriptive study with ethnographic methodology using semi-structured interviews	9 men from Senegal and Mali, living in Spain, average age 35	To identify perspective of men on A/FGM**** to increase the cultural understanding of factors that support this practice	The results show that the A/FGM was conceived as a system of traditional cares subtended by several sexual, hygienic and religious factors. FGM was considered to guarantee faithfulness of wives in marriage and hence plays an important role in the maintenance of polygamy
Sagna (2014) [40]	Sierra Leone	DHS data of Sierra Leone, 2008	3123 men aged 15-59	To determine men’s and women’s attitude toward discontinuation of FGC	More men (36 %) compared to women (25 %) thought there were no benefits for a girl to undergo FGC. Men who saw no benefit had four times odds of supporting discontinuation. Men were significantly less likely to support discontinuation if they saw it as religious requirement.
Sakeah et al. (2006) [30]	Ghana	Simple random sample technique in a district of northern Ghana; questionnaire survey with two parts: part one to all men and part two to only those who had heard of FGM	1,406 men aged 12-24, 1,114 men aged 25 and above	To determine factors associated with men’s preference for circumcised women	18.8 % of men preferred women who had FGC. Men who preferred women with FGC were significantly more likely to be illiterate or have only primary or middle school education as compared to secondary and higher education. Men who preferred women to have FGC were significantly more likely to be Nankana compared to Kassesa ethnicity and more likely to report their religion as traditional compared to Christianity.

*female genital cutting

**female genital mutilation/cutting

*** female circumcision

****ablation/female genital mutilation

## Results

Twenty peer-reviewed articles were included in our analysis. Nine were quantitative surveys [22–30], ten used qualitative interviews [31–40] and one was a mixed qualitative and quantitative study [41]. The settings included 15 countries, i.e. Egypt, Yemen, Oman, Nigeria, North Sudan, Senegal, Guinea, Somalia, Gambia, Sierra Leone, Ghana, USA, Norway, Sweden and Spain.

Three main themes in regards to men’s attitudes, beliefs and behaviours to support continuation or abandonment of FGM and its prevention emerged. These were (1) men’s perceptions of FGM, (2) FGM as an issue for men, and (3) influence of socio-demographic factors. A synthesis of the available data revealed ambiguity of men’s wishes in regards to the continuation of FGM. Many men wished to abandon this practice because of the physical and psychosexual complications to both women and men. The silent culture between the sexes was posited as a major obstacle for change [32], as was the entrenched sense of social obligation [31, 35, 37].

### Men’s perception of FGM

A study of fathers in Egypt showed that they believed uncut women to be promiscuous [31]. FGM was deemed important for good marriage opportunities and to ensure fidelity in marriage [31]. In this respect, FGM helped men maintain polygamy in some communities [39]. Men in Guinea considered FGM to reduce the likelihood of premarital sex [24]. In a study of Somali men, however, they were divided on whether FGM prevented premarital sex, marital infidelity and preserved the dignity of girls [25].

Men acknowledged and complained about the negative impact of FGM on marital sexual relationships, and found the lack of sexual response of their wives disturbing or inconvenient [31, 33]. Almost all 99 men and religious leaders, Muslims and Christians, in a study in rural communities in Egypt acknowledged women’s equal right to enjoy sex [33]. Nevertheless, for some men these concerns and beliefs were overridden by their wish to ensure their wives’ fidelity in marriage [31] or their fear of loss of control over the sexual relationship [33].

### FGM as an issue for men

Interviews with men in Northern Sudan revealed that men did not accurately understand FGM, as it was not until they were newly married that they experienced the irrevocable consequences of their wives’ FGM [32]. Men felt they, too, were victims of the consequences of FGM. Almost all men stated they did not want their daughters to undergo FGM and believed it would become less common as men had started to prefer women who had not been cut [32]. Men described their own complications, including male sexual dissatisfaction, compassion for female suffering and perceived challenges to their masculinity [32, 33].

### Factors that influence men’s support for continuation or abandonment of FGM

#### Social obligation

Somali men in Oslo acknowledged that men in Somalia disliked the practice but that it continued due to social obligation [35]. Men agreed to it so as not to upset their mothers [37]. Somali men in Norway no longer felt social pressure to perform FGM. In fact, they maintained that it was prestigious for a woman not to have been cut [35].

Fathers in Egypt acknowledged the wish to abandon FGM and a longing for change [31]. They cited social pressure and fear of rejection from the community as significant barriers to the abandonment process. The entrenched sense of social obligation was stronger than the belief that FGM was against their religion [31].

### Education, urban living, religion and ethnicity

The level of education of men, urban living and wealth are associated with disapproval of FGM [24, 26, 29, 30]. Evaluation of DHS data in Guinea from 1999 revealed that 51 % of men wanted FGM to continue, whilst 38 % were against it [24]. Each additional year of schooling substantially increased the odds of favouring the discontinuation of the practice [24].

A school-based study of adolescent boys in Oman revealed that they were more likely to support FGM if they lived in rural areas and their parents had lower level of education [26]. Eighty percent of the boys considered FGM to be important and necessary.

The analysis of the DHS of Guinea showed that if FGM was considered to be accepted by religion, men were more likely to be supportive of the practice [24]. In two studies in Somalia, almost all men supported the continuation of FGM and 96 % preferred to marry women who had been cut, even though 90 % were aware of its complications [25, 34]. Men supported the “lesser” Sunna type, i.e. types I and II, because they believed it not to have any negative health effects, unlike the Pharaonic type, i.e. type III or infibulation [8]. Ninety-six percent of men believed FGM to be a religious requirement.

Prevalence of FGM varied amongst Muslims with different ethnic backgrounds from 12 % to 98 % in a study of 993 men in Gambia [38]. The Serer and Wolof communities that were Muslim but traditionally non-practising, had the lowest prevalence. Wolof men also had the highest awareness of complications of FGM [38]. Similarly, male healthcare workers in Gambia belonging to traditionally practising communities were more likely to support the continuation and medicalisation of FGM, and intended to cut their daughters [38].

### Knowledge of complications of FGM

Intervention studies involving men had an important positive effect on men’s attitudes towards abandonment of this practice. In a study of men (*n* = 4488) and women (*n* = 5041) in Nigeria [41], a greater proportion of men (54 %) than women (44 %) did not want FGM stopped prior to the intervention of health education on FGM and its complications over ten days. There was a statistically significant decrease in this attitude to 25 % amongst men in the post-intervention stage.

A six months Village Empowerment Program was conducted by TOSTAN in Senegal on human rights, problem-solving process, basic hygiene, and women’s health [23]. The change in the intention to cut their daughters amongst men was greatest among program participants (66 to 13 %) and least in the control group (78 to 56 %). Twenty percent of men as participants and 63 % in the comparison groups preferred a women who had been cut. Most participant men (75 %) indicated their support for the abandonment of FGM. Only 30 % in the comparison group expressed the same.

In a study of 993 men in Gambia, 72 % did not know FGM had a negative impact on health [38]. As compared to older men, younger men had a better understanding of the health problems and were less supportive of the practice, had lower intention to cut their daughters, and had higher willingness for men to participate in prevention programs [38].

### Migration

There are three studies that examined the attitudes of men from Somalia in Norway [35] and the USA [37], and from Ethiopia and Eritrea in Sweden [36]. In contrast to findings from countries where FGM is prevalent, almost all men strongly rejected this practice [35–37]. Men had very good knowledge of the complications of FGM [35–37] and understood that it reduced female sexual pleasure [35, 36]. They considered it devoid of meaning within the context of a cultural practice and that it had no religious mandate [35, 36]. One man had believed it was done to girls to prevent sexual violence [35].

Even living in another African country had a positive effect on attitudes of men. Eighty-nine percent of Somali male refugees in Ethiopia positively viewed the usefulness of anti-FGM interventions [28].

## Discussion

Our systematic review supports the two main factors perpetuating the continuation of FGM, namely social obligation and marriageability [1]. The former relates to social pressure to adhere to norms, which vary among different communities and countries. The norms may pertain to perceived religious requirement, family honour through premarital virginity of daughters and marital fidelity of wives, aesthetics, and rite of passage [42–44]. Fear of exclusion from resources and opportunities as a young woman, including a good marriage, are other important reasons [42, 43]. Men may play a passive role in approving FGM by refusing to marry uncut women or an active one by initiating the practice [9]. In a study of about 400 Nigerian men and women, 71 % of them stated that it was paternal grandfathers and fathers who were the decision makers responsible for requesting FGM [45].

On the other hand, many men wish the practice to end but are unable to voice their concerns. In Guinea, Sierra Leone and Chad, for example, more men than women want FGM to end [1]. There is evidence from DHS data that there may be limited dialogue on FGM between the genders [1]. In some surveys, women and girls tended to consistently underestimate the proportion of men and boys who wanted FGM to end. Similarly, in some surveys many women and men did not know the opinion of the opposite sex in regards to FGM [1]. Enabling communication between men and women, as well as among men, and opening up this practice to a debate of its validity in a culturally sensitive way warrants further research and may facilitate the abandonment process. In a family planning study, teaching communication skills to men to facilitate conversations on contraception with their partners, not only increased contraception uptake but also improved spousal relationships [46].

Our review suggests that FGM affects men as well as women and that it can no longer be considered an issue pertaining only to women’s health [3]. Men married to women with FGM have health complications as well and feel they, too, are victims of this practice [32]. Indeed, the adverse effects of FGM on men have been well documented in a Sudanese study of married men (*n* = 59), most of whom expressed difficulty with vaginal penetration, wounds or infections on the penis and psychosexual problems [3]. Most notable was the finding that men perceived their wives’ suffering as their own problem. Most of the young men stated they would have preferred to be married to uncut women [3].

Our results reveal that education, age, knowledge of the health complications of FGM, religion, urban living, ethnicity, and migration influence men’s stated support for the abandonment of this practice. These findings are in keeping with the UNICEF 2013 report of analysis of DHS data over 20 years from 29 countries of Africa and the Middle East [1]. The common thread that binds these factors is education. Involvement of men in sexual and reproductive health promotion, for example, has been a successful strategy to help women with family planning, HIV/STI prevention, violence against women, and maternity care [46–50]. Studies have shown that men do want to be involved, and respond positively to efforts to involve them in these programs, as they care about the welfare of their families [51, 52]. A study in Nepal, for instance, showed that educating pregnant women and their male partners had a greater impact on maternal health behaviours compared with educating women alone [47]. The relationship between education level and support for the abandonment of FGM, however, is presented through bivariate analysis and further research through multivariate analysis would help to determine causality.

Involvement of men in reproductive health services to date has been with the sole purpose of benefit to women [48]. In a study of male involvement in maternity health care in Malawi, men felt they were not the beneficiaries and were merely used as a means to get women to the health service [53]. Moreover, due to gender dynamics, men attending women’s clinics with their wives were vulnerable and ridiculed by other men [54]. A more positive and successful involvement of men in the abandonment of FGM hence may be achieved by the provision of reproductive health services specific for men. A man-to-man strategy would allow open discussion of private and sensitive health and other personal men’s issues. Men also, like women, need to be empowered through health literacy to be able to make informed and healthy decisions for themselves and their families. Interviews of Kenyan men suggested men-only community groups for creating awareness and conducting male reproductive health education [54]. Education has also been achieved through schools, social media, mobile phone technology, sporting events, musicians, radio, theatre and puppet shows [54, 55]. Male musicians or sportsmen themselves could be key advocates for the abandonment of FGM. Using videos depicting graphic images of the practice has been particularly effective with men who became aware of the suffering involved for the first time [55].

It may be beneficial for the abandonment process if men’s intervention and education programs worked with those of women’s. Our study shows that some men distinctly wish the harmful practice of FGM to continue even if they believed their religion did not condone it. Their self-interest is to support polygamy in some communities and control the sexuality of their wives. This requires opposition and a voice from women. It requires their financial empowerment through education and independence from men.

In our review, some men highlighted that change should come from within their own community rather than governments or nongovernment organisations [34]. Communities in Sub-Saharan Africa endure many human rights abuses in addition to FGM, such as lack of access to clean water, food security, health services and education, child marriage, and sexual violence [56]. Addressing communities’ priorities would be an important gateway to earning their trust and working with men and women towards the abandonment of FGM. This is borne out by our review that migration is a positive influence to the abandonment of FGM. We may speculate on the reasons for this phenomenon. When people are granted their basic human rights with stable and improved social and economic living options, the need to cut their daughter for marriageability and economic survival is removed. Moreover, social pressure is relieved, as FGM is counter-normative in the new country. Instead of FGM accruing positive outcomes like a good marriage, it causes prejudice and disadvantage, and becomes a liability. As borne out by the study of Somali migrants in Norway [35], uncut Somali girls were more likely to attract boyfriends and get married as compared to girls who had been subjected to FGM.

## Strengths and limitations

This study is the first in the literature to present a systematic review of the role of men in FGM. It provides evidence on the importance of and need for directing research and intervention programs to involve men in the abandonment process. The limitations pertain mainly to measurement, interviewer, and response biases in the studies. In FGD especially, men may be reluctant to give socially unacceptable answers for a topic that has such high social pressure for conformity.

In particular, in the intervention studies, subjects may have acknowledged to the interviewer that they did not support the continuation of FGM at endline because they believed this to be the answer they wanted to hear. In some studies, money was given to subjects for participation, which introduced selection and response bias. FGM is a prosecutable offence in most of the countries where it is performed. Hence, in the studies cited, men may not have felt they could freely disclose their beliefs. The overall findings of the review cannot be generalised to all men in regards to FGM, as prevalence, views and behaviours are specific to countries and communities. Moreover, even though men’s opinions are stated and they may support abandonment, we do not know their influence on the decision making process to subject girls to FGM.

## Conclusion

Men have conflicting views on FGM. Many would like it to end but are unable to voice their support for its abandonment due to social pressure and obligation within the community. Change needs to come from within communities, supported by the creation of opportunities for men and women to debate the practice amongst themselves. Advocacy by men, as well as research, prevention programs and health services targeted at men could be explored to assess their success within the abandonment process. These programs may work together with those for women to empower men and women to decide to abandon this harmful practice to protect their daughters, men and communities from the devastating effects of this harmful practice.

## Abbreviations

FGM: Female genital mutilation
USA: United States of America
PICOS: Population, Interventions, Comparison, Outcomes, Study design
PRISMA: Preferred Reporting Items for Systematic Reviews and Meta-Analyses
CINAHL: Cumulative Index to Nursing and Allied Health
FGM: Female genital cutting
RI: Re-infibulation
FGM/C: Female genital mutilation/cutting
FC: Female circumcision
A/FGM: Ablation/female genital mutilation
DHS: Demographic and Health Surveys
UNICEF: United Nations Children’s Fund
HIV/STI: Human immunodeficiency virus/sexually transmitted infections
FGD: Focus group discussion(s)

## References

[1] Female genital mutilation/cutting: A statistical overview and exploration of the dynamics of change, UNICEF, New York [Internet]. 2013 July. Available from: http://www.unicef.org/publications/index_69875.html. Accessed 16 Jun 2015.

[2] Female genital mutilation/cutting: What might the future hold? [Internet]. 2014. Available from: http://reliefweb.int/sites/reliefweb.int/files/resources/FGM-C_Report_7_15_Final_LR.pdf. Accessed 16 Jun 2015.

[3] Almroth L, Almroth-Berggren V, Hassanein OM, Al-Said SSE, Hasan SSA, Lithell U-B (2001). Male complications of female genital mutilation. Soc Sci Med.

[4] World Health Organization. Sexual and Reproductive Health. Classification of female genital mutilation [Internet]. 2008. Available from: http://www.who.int/reproductivehealth/topics/fgm/overview/en/index.html. Accessed 16 Jun 2015.

[5] Almroth L, Bedri H, El Elmusharaf S, Satti A, Idris T, Hashim MSK (2005). Urogenital complications among girls with genital mutilation: A hospital based study in Khartoum. Afr J Reprod Health.

[6] Talle A, Hernlund Y, Shell-Duncan B (2007). Female circumcision in Africa and beyond: the anthropology of a difficult issue. Transcultural bodies: female genital cutting in global context.

[7] Elnashar RA, Abdelhady R (2007). The impact of female genital cutting on health of newly married women. Int J Gynaecol Obstet.

[8] World Health Organization. Eliminating female genital mutilation: An interagency statement–OHCHR, UNAIDS, UNDP, UNECA, UNESCO, UNFPA, UNHCR, UNICEF, UNIFEM, WHO [Internet]. 2008. Available from: http://www.who.int/reproductivehealth/publications/fgm/9789241596442/en/. Accessed 16 Jun 2015.

[9] Davis G, Ellis J, Hibbert M, Perez RP, Zimbelman E (1999). Female circumcision: the prevalence and nature of the ritual in Eritrea. Mil Med.

[10] Missailidis K, Gebre-Medhin M (2000). Female genital mutilation in Eastern Ethiopia. Lancet.

[11] Broadwin J (1997). Walking contradictions: Chinese women unbound at the turn of the century. J Hist Sociol.

[12] Lucas PJ, Baird J, Arai L, Law C, Roberts HM (2007). Worked examples of alternative methods for the synthesis of qualitative and quantitative research in systematic reviews. BMC Med Res Methodol.

[13] Tacconelli E (2010). Systematic reviews: CRD’s guidance for undertaking reviews in health care. Lancet Infect Dis.

[14] Moher D, Liberati A, Tetzlaff J, Altman DG (2009). Preferred reporting items for systematic reviews and meta-analyses: the PRISMA statement. Ann Intern Med.

[15] Shell-Duncan B, Wander K, Hernlund Y, Moreau A (2011). Dynamics of change in the practice of female genital cutting in Senegambia: testing predictions of social convention theory. Soc Sci Med.

[16] Merli C (2010). Male and female genital cutting among Southern Thailand’s Muslims: rituals, biomedical practice and local discourses. Cult Health Sex.

[17] Scorgie F, Beksinska M, Chersich M, Kunene B, Hilber AM (2010). Smit J: “Cutting for love”: genital incisions to enhance sexual desirability and commitment in KwaZulu-Natal, South Africa. Reprod Health Matter.

[18] Critical Appraisal Skills Programme. 10 questions to help you make sense of qualitative research [Internet]. 2013. Available from: http://media.wix.com/ugd/dded87_29c5b002d99342f788c6ac670e49f274.pdf. Accessed 16 Jun 2015.

[19] Canadian National Collaborating Centre for Methods and Tools. Quality assessment tool for quantitative studies [Internet]. 2008. Available from: http://www.nccmt.ca/registry/view/eng/14.html. Accessed 16 Jun 2015.

[20] Ali C (2012). Strøm A: “It is important to know that before, there was no lawalawa.” Working to stop female genital mutilation in Tanzania. Reprod Health Matter.

[21] Harden A, Garcia J, Oliver S, Rees R, Shepherd J, Brunton G (2004). Applying systematic review methods to studies of people’s views: an example from public health research. J Epidemiol Commun H.

[22] Al-Khulaidi GA, Nakamura K, Seino K, Kizuki M (2013). Decline of supportive attitudes among husbands toward female genital mutilation and its association to those practices in Yemen. PLoS One.

[23] Diop NJ, Askew I (2009). The effectiveness of a community‐based education program on abandoning female genital mutilation/cutting in Senegal. Stud Family Plann.

[24] Gage AJ, Van Rossem R (2006). Attitudes toward the discontinuation of female genital cutting among men and women in Guinea. Int J Gynecol Obstet.

[25] Gele AA, Bø BP, Sundby J (2013). Have we made progress in Somalia after 30 years of interventions? Attitudes toward female circumcision among people in the Hargeisa district. BMC Res Notes.

[26] Jaffer YA, Afifi M, Al Ajmi F, Alouhaishi K (2006). Knowledge, attitudes and practices of secondary-school pupils in Oman: II. Reproductive health. East Mediterr Health J.

[27] Kaplan A, Hechavarria S, Bernal M, Bonhoure I (2013). Knowledge, attitudes and practices of female genital mutilation/cutting among health care professionals in The Gambia: a multiethnic study. BMC Public Health.

[28] Mitike G, Deressa W (2009). Prevalence and associated factors of female genital mutilation among Somali refugees in eastern Ethiopia: a cross-sectional study. BMC Public Health.

[29] Ouldzeidoune N, Keating J, Bertrand J, Rice J (2013). A description of female genital mutilation and force-feeding practices in Mauritania: implications for the protection of child rights and health. PLoS One.

[30] Sakeah E, Beke A, Doctor HV, Hodgson AV (2006). Males’ preference for circumcised women in Northern Ghana. Afr J Reprod Health.

[31] Abdelshahid A, Campbell C (2015). Should I circumcise my daughter? Exploring diversity and ambivalence in Egyptian parents’ social representations of female circumcision. J Community Appl Soc Psychol.

[32] Berggren V, Ahmed SM, Hernlund Y, Johansson E, Habbani B, Edberg A-K (2006). Being victims or beneficiaries? Perspectives on female genital cutting and reinfibulation in Sudan. Afr J Reprod Health.

[33] Fahmy A, El-Mouelhy MT, Ragab AR (2010). Female genital mutilation/cutting and issues of sexuality in Egypt. Reprod Health Matters.

[34] Gele AA, Bente PB, Sundby J (2013). Attitudes toward female circumcision among men and women in two districts in Somalia: is it time to rethink our eradication strategy in Somalia?. Obstet Gynecol Int.

[35] Gele A, Kumar B, Hjelde K, Sundby J (2012). Attitudes towards female circumcision among Somali immigrants in Oslo: a qualitative study. Int J Womens Health.

[36] Johnsdotter S, Moussa K, Carlbom A, Aregai R, Essén B (2009). “Never my daughters”: A qualitative study regarding attitude change toward female genital cutting among Ethiopian and Eritrean families in Sweden. Health Care Women Int.

[37] Johnson-Agbakwu CE, Helm T, Killawi A, Aasim I (2014). Perceptions of obstetrical interventions and female genital cutting: insights of men in a Somali refugee community. Ethn Health.

[38] Kaplan A, Cham B, Njie L, Seixas A, Blanco S, Utzet M: Female genital mutilation/cutting: the secret world of women as seen by men. Obstet Gynecol Int, 2013, Article ID 643780, 11 pages, 2013. doi:10.1155/2013/643780. 10.1155/2013/643780 PMC3723317 23935631

[39] Ruiz IJ, Bravo MDMP, Martínez PA, Meseguer CB (2014). Men facing the ablation/female genital mutilation (A/FGM): cultural factors that support this tradition. Procedia Soc Behav Sci.

[40] Sagna ML (2014). Gender differences in support for the discontiuation of female genital cutting in Sierra Leone. Cult Health Sex.

[41] Asekun-Olarinmoye EO, Amusan OA (2008). The impact of health education on attitudes towards female genital mutilation (FGM) in a rural Nigerian community. Eur J Contracept Reprod Health Care.

[42] United Nations Children’s Fund (UNICEF). The dynamics of social change–Towards the abandonment of female genital mutilation/cutting in five African countries. Florence, Italy: UNICEF Innocenti Research Centre. [Internet]. 2010. Available from: http://www.unicef-irc.org/publications/pdf/fgm_insight_eng.pdf. Accessed 16 Jun 2015.

[43] United Nations Children’s Fund (UNICEF). Changing a harmful social convention: Female genital mutilation/cutting in five African countries. In: Innocenti Digest. Florence: Innocenti Research Centre. [Internet]. 2007. Available from: http://pages.ucsd.edu/~gmackie/documents/ChangingHarmfulSocialConvention.pdf. Accessed 16 Jun 2015.

[44] World Health Organization (WHO). An update on WHO’s work on female genital mutilation (FGM): progress report. Geneva: Dpt of Reproductive Health and Research. [Internet]. 2011. Available from: http://www.who.int/reproductivehealth/publications/fgm/rhr_11_18/en/. Accessed 16 Jun 2015.

[45] Amusan OA, Asekun-Olarinmoye EO (2006). Knowledge, beliefs, and attitudes to female genital mutilation (FGM) in Shao Community of Kwara State, Nigeria. Int Q Community Health Educ.

[46] Shattuck D, Kerner B, Gilles K, Hartmann M, Ng’ombe T, Guest G (2011). Encouraging contraceptive uptake by motivating men to communicate about family planning: the Malawi Male Motivator project. Am J Public Health.

[47] Mullany BC, Becker S, Hindin MJ (2007). The impact of including husbands in antenatal health education services on maternal health practices in urban Nepal: results from a randomized controlled trial. Health Ed Res.

[48] Sternberg P, Hubley J (2004). Evaluating men’s involvement as a strategy in sexual and reproductive health promotion. Health Promot Int.

[49] Adongo PB, Tapsoba P, Phillips JF, Tabong PTN, Stone A, Kuffour E (2013). The role of community-based health planning and services strategy in involving males in the provision of family planning services: a qualitative study in Southern Ghana. Reprod Health.

[50] Population Council. Mixed success involving men in maternal care worldwide. Population Briefs. Reports of Population Council Research 2005 [Internet]. Available from: http://www.popcouncil.org/uploads/pdfs/pbjan05.pdf. Accessed 16 Jun 2015.

[51] Baylies C, Bujra J (2000). AIDS, Sexuality and Gender in Africa; the Struggle Continues; Collective Strategies for Protection Against AIDS in Tanzania and Zambia.

[52] Drennon M (1999). Reproductive Health. New Perspectives on Men’s Participation. Population Reports Series J, Family planning progams.

[53] Kululanga LI, Sundby J, Malata A, Chirwa E (2012). Male involvement in maternity health care in Malawi: original research article. Afr J Reprod Health.

[54] Onyango MA, Owoko S, Oguttu M (2010). Factors that influence male involvement in sexual and reproductive health in Western Kenya: a qualitative study: original research article. Afr J Reprod Health.

[55] Spadacini B, Nichols P (1998). Campaigning against female genital mutilation in Ethiopia using popular education. Gender and Development.

[56] Varol N, Fraser IH, Ng C, Jaldesa G, Hall J (2014). Female genital mutilation/cutting: towards abandonment of a harmful traditional practice. Aust NZ J Obstet Gynaecol.

